# The genetic diversity and population structure of *Sophora alopecuroides* (Faboideae) as determined by microsatellite markers developed from transcriptome

**DOI:** 10.1371/journal.pone.0226100

**Published:** 2019-12-05

**Authors:** Yuan Wang, Tingting Zhou, Daihan Li, Xuhui Zhang, Wanwen Yu, Jinfeng Cai, Guibin Wang, Qirong Guo, Xiaoming Yang, Fuliang Cao

**Affiliations:** Co-Innovation Center for Sustainable Forestry in Southern China, Nanjing Forestry University, Nanjing, China; Beijing Forestry University, CHINA

## Abstract

*Sophora alopecuroides* (Faboideae) is an endemic species, mainly distributed in northwest China. However, the limited molecular markers range for this species hinders breeding and genetic studies. A total of 20,324 simple sequence repeat (SSR) markers were identified from 118,197 assembled transcripts and 18 highly polymorphic SSR markers were used to explore the genetic diversity and population structure of *S*. *alopecuroides* from 23 different geographical populations. A relatively low genetic diversity was found in *S*. *alopecuroides* based on mean values of the number of effective alleles (*N*_e_ = 1.81), expected heterozygosity (*H*_e_ = 0.39) and observed heterozygosity (*H*_o_ = 0.55). The results of AMOVA indicated higher levels of variation within populations than between populations. Bayesian-based cluster analysis, principal coordinates analysis and Neighbor-Joining phylogeny analysis roughly divided all genotypes into four major groups with some admixtures. Meanwhile, geographic barriers would have restricted gene flow between the northern and southern regions (separated by Tianshan Mountains), wherein the two relatively ancestral and independent clusters of *S*. *alopecuroides* occur. History trade and migration along the Silk Road would together have promoted the spread of *S*. *alopecuroides* from the western to the eastern regions of the northwest plateau in China, resulting in the current genetic diversity and population structure. The transcriptomic SSR markers provide a valuable resource for understanding the genetic diversity and population structure of *S*. *alopecuroides*, and will assist effective conservation management.

## Introduction

*Sophora alopecuroides* L. (Faboideae, Leguminosae), known as kudouzi in China, is a perennial herb in the genus *Sophora* (2N = 36). As a wild and moderately xerophytic species, it is mainly distributed in the arid and semi-arid regions of the Asian continent, particularly in the northwest plateau in China [1]. The fully developed root system of *S*. *alopecuroides* confers its excellent drought and alkaline tolerance and anti-sandstorm performance, making it a vital pioneer in environmental protection in northwest China. It is noteworthy for its pharmaceuticals and pesticidal properties, as well as being a valuable source of livestock forage, green manure, windbreaks and nectar [1]. Furthermore, *S*. *alopecuroides* is also fine forage for increasing the weight of livestock. According to records in the well-known and comprehensive medical book *Compendium of Materia Medica* from the Ming dynasty in China, it has been used as traditional Chinese medicine for thousands of years. It possesses high concentrations of secondary metabolites, especially alkaloids, flavonoids and triterpenoids, which exhibit anti-inflammatory, anti-viral and anti-tumor activities [2, 3], and it has been proven to treat fever, diarrhea and even to inhibit cancer cells [2].

Assessment of genetic diversity is a prerequisite for efficiently exploring and utilizing germplasms, and developing different conservation approaches to breeding [4]. Recently, a wide variety of genetic markers including as allozyme, amplified fragment length polymorphisms, chloroplast DNA sequences, simple sequence repeats and single nucleotide polymorphism and insertion-deletion, has been used to investigate levels of genetic diversity and population structure in Faboideae [5–10]. Despite its medicinal value and ecological importance, however, current researches involving *S*. *alopecuroides* focuses mainly on its chemical constituents, pharmacological activity, and endophytic resources [11–13], while molecular research lags behind seriously. A comprehensive study is urgently needed for an in-depth understanding of the genetic diversity and population structure of *S*. *alopecuroides* to clarify implications for medicine resource exploitation and ecology conservation.

SSR markers are regarded as one of the most powerful high-resolution tools for the study of population genetics, phylogenetics, molecular ecology and marker-assisted selection studies in plants for characteristics of codominance, good reproducibility and high polymorphism [14, 15]. The rapid advance in RNA-sequencing have enabled genome-wide discovery of thousands of SSRs in various species, especially for non-model organisms lacking of more genome information [16–18]. Transcriptional analysis, novel gene discovery and molecular marker development has been extensively explored by RNA-sequencing in Faboideae, such as chickpea (*Cicer arietinum*) [19], winged bean (*Psophocarpus tetragonolobus*) [20] and black locust (*Robinia pseudoacacia*) [21]. However, RNA-sequencing has not been applied to devise appropriate breeding schemes or to curry out population genetic structure researches on *S*. *alopecuroides*. There was a substantial lack of available genomic information, genetic linkage map and polymorphism molecular markers, which had limited genetic researches on *S*. *alopecuroides*.

To our knowledge, the present study reports the first transcriptome sequencing of *S*. *alopecuroides*. The primary goal of the study was to evaluate the genetic diversity and population structure with a large collection of germplasms based on SSR markers. Specifically, our investigations aimed to: (1) characterize the transcriptome of *S*. *alopecuroides*; (2) develop and validate a series of SSR markers to assess the genetic diversity and elucidate genetic relationships and population structure among different populations; and (3) propose effective conservation strategies for the valuable *S*. *alopecuroides*.

## Materials and methods

### Ethics statement

Our research did not involve the rare and endangered plants. Samples of *S*. *alopecuroides* were not gathered from any nature reserve or private land. Therefore, collection of the material did not require an additional approval of the ethics committee or other specific permission.

### Plant materials and DNA isolation

**Fig 1:**
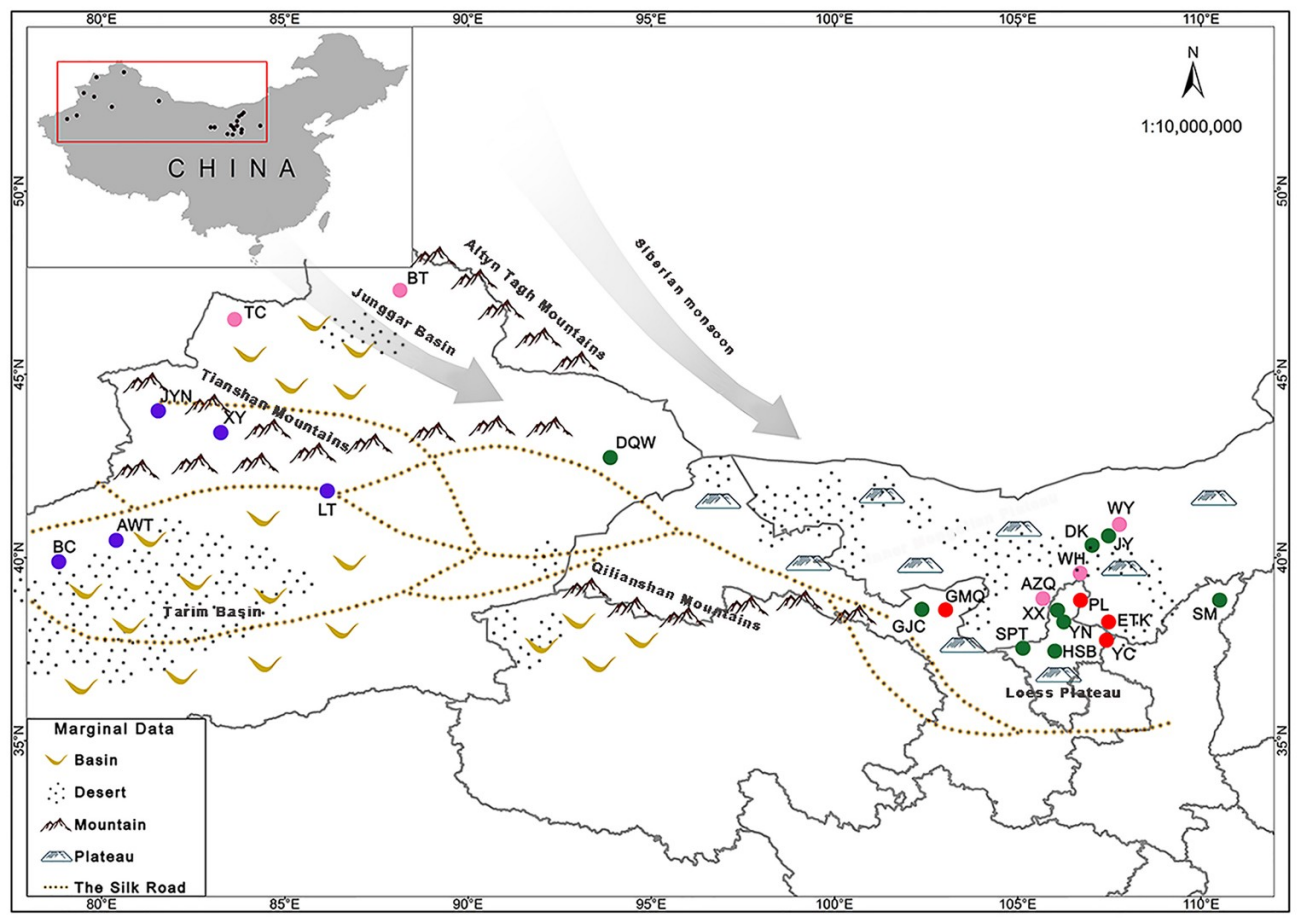
Geographic distribution of 23 populations of *S*. *alopecuroides* from the northwest China. Circles with colors denote the position of sampling sites. Cluster I, cluster II, cluster III and Cluster IV are presented by blue, pink, red and green circles, respectively.

A total of 260 individual plants were collected from 23 widely spread natural populations covering the majority of the geographical range of *S*. *alopecuroides* (Fig 1 and S1 Table) in northwest China. Only fresh and healthy plants were collected, chosen at random and ensuring that the distance between them was at least 100m. The number of individuals sampled in some populations was less than 10 owing to their limited population size. After rapid drying using silica gel, total genomic DNA was isolated from leaf materials using a plant genome DNA isolation kit (Bioteke, China) following the manufacturer’s instructions. The concentration and quantity of DNA was tested with NanoDrop 2000 (Thermo Fisher Scientific Inc., Waltham, MA, USA).

### Transcriptome sequencing, de novo transcriptome assembly and function annotation

Total RNA was isolated from young fresh leaves, stems and roots of *S*. *alopecuroides* using the Trizol reagent (Invitrogen Life Technologies, USA) according to the manufacturer’s instructions. Pooled samples were prepared by combining equimolar concentration of total RNA for each of three tissues. The integrity of the qualified RNA was analysed through agarose gel electrophoresis and the quality of the total RNA was checked on a NanoDrop 2000 spectrophotometer (Thermo Scientific). The cDNA library for mRNA-seq was prepared with high quality RNA of pooled samples using Illumina Truseq^™^ RNA sample prep Kit (Illumina, CA, USA). The cDNA libraries were sequenced using the Illumina HiSeq^™^ 2500 platform using the paired-end model to obtain read lengths of 150 bp. Sequenced raw data described in this study were uploaded in the Sequence Read Archive (SRA) public database of the NCBI (PRJNA546451).

Prior to assembly, the raw paired-end reads were submitted to quality filtering and adaptor trimming using Trimmomatic version 0.36 [22]. Clean reads were obtained from the raw sequencing reads by removing all reads with adaptor sequences, low-quality reads (reads with more than 50% of the base mass values less than 5), as well as ambiguous sequences containing in excess of 10% unknown nucleotides. The de novo assembly of the transcriptome was accomplished using all the clean reads and Trinity version 2.6.6 [23] using the deBruijn graph method with default settings.

The generated non-redundant unigenes were annotated to public databases using aligned BLASTX [24] with several databases (E < 1e^-5^) including Pfam protein, Kyoto Encyclopedia of Genes and Genomes (KEGG), Clusters of Orthologous Group (COG), Swiss-Prot protein, the NCBI non-redundant protein (Nr) and non-redundant nucleotide sequences (Nt) to predict possible functional classifications and signaling pathways. Gene ontology (GO) annotation was implemented on Blast2GO [25, 26] and then plotted with functional classification using WEGO version 2.0 [27].

### Detection of EST-SSR markers and primer design

All unigenes generated by deep transcription sequencing of *S*. *alopecuroides* were screened for SSRs using the Perl script MISA (https://webblast.ipk-gatersleben.de/misa/). A minimum of ten repeats for mononucleotide SSRs, a minimum of six repeats for dinucleotide SSRs, and a minimum of five repeats for tri-, tetra-, penta- and hexanucleotide were considered as the search criteria for simple sequence repeat in the MISA script. Primer pairs were designed using Primer3 (http://primer3.sourceforge.net/) with the following criteria: annealing temperature of 50–60 °C, optimum primer length of 20–24 bp, a percentage GC in the range of 40%–60%, and predicted product size of 100–400 bp.

### Genetic diversity and population structure analysis

Each PCR amplification was performed with 1.1 × T3 Super PCR Mix (Tsingke, China) in a final volume of 20 μl, containing 50 ng DNA, 1 μl each of 10 ng forward and reverse primers. PCR reactions were carried out in the following steps: 5 min pre-denaturation at 94 °C, 35 cycles at 94 °C for 45 s, annealing at an optimal temperature ranging from 55 °C to 60 °C, extension at 72 °C for 45 s, then a final extension at 72 °C for 10 min. Each of the PCR products was confirmed using 6% polyacrylamide gel electrophoresis. We treated these microsatellites as codominant marker when we conducted the following analyses. The genetic diversity indexes including number of alleles (*N*_a_), the number of effective alleles (*N*_e_), number of rare allele (*N*_r_), number of unique allele (*N*_u_), polymorphic information content (PIC), observed heterozygosity (*H*_o_), and expected heterozygosity (*H*_e_), fixation index (*F*_is_), and information index (*I*), Hardy-Weinberg equilibrium were estimated using GenAlEx version 6.5 [28] and Arlequin version 3.5 [29].

Population structure analysis was assessed with STRUCTURE version 2.3 [30] based on the individual-based Bayesian clustering methods. A continuous series of *K* values from 1 to 10 were tested in 10 independent runs to deduce the optimal *K* value for the genotypes. Each run comprised a burn-in length of 100,000 followed by 1,000,000 MCMC (Monte Carlo Markov Chain) replicates. The most likely values of *K* were chosen based on *ΔK* that computed with Structure Harvester [31]. The program CLUMPP version 1.1.2 [32] with the greedy algorithm and 10,000 random input orders of 10 independent STRUCTURE runs to determine the optional alignment of clusters across individual runs for each *K*. The genetic structure plot was drawn by Distruct version 1.1 [33]. To reveal population structure based on the genetic distance among all sampled individuals, principal component analysis (PCoA) was performed with GenAlEx version 6.5. A Neighbor-Joining (NJ) tree based on Kimura’s two-parameter distances was also constructed with MEGA version 6.0 [34].

An analysis of molecular variance (AMOVA) and estimate of pairwise *F* statistics (*F*_st_) among the groups were performed using Arlequin version 3.5 to measure the probable differentiation among different groups.

### Geographic and genetic correlations, and bottleneck effect analysis

Mantel tests were conducted based on genetic distance (*F*_st_ / (1 –*F*_st_)) and geographic distance (km) in GenAlEx version 6.5. A heterozygosity excess test at the population level from BOTTLENECK 1.2.02 [35, 36] was used to detect a recent population bottleneck. A two-phase model (TPM) was set up using 95% of the single-step stepwise mutation model (SMM) and 5% of the infinite allele model (IAM) with a variance of 12 among multiple steps [36]. Significance was tested using Wilcoxon’s signed-rank test under the assumption of mutation-drift equilibrium [35] and a mode-shift test was also used to detect genetic bottlenecks.

## Results

### De novo assembly and sequence annotation

The Illumina sequencing generated a total of 390,247,286 clean reads after adaptor removal. The length of assembled sequences varied from 200 bp to more than 16,303 bp, with the average of approximately 735 bp, GC content was approximately 47.96%, and the length of N50 was 1,213 bp (S2 Table). After sequence contig assembly, 78,368 assembled unigenes in total were annotated by comparison against seven functional databases. A total of 36,049 (46.00%), 30,227 (38.57%), 20,310 (25.92%), 23,035 (29.39), 23,248 (29.67%), 10,680 (13.63%), and 26,332 (33.6%) unigenes showed homology to available sequences in Nr, Nt, KOG, SwissProt, GO, KEGG and Pfam databases, respectively. Taken together, of total unigenes, 52.7% of unigenes were found a match to any one of the databases and 7.96% were annotated in all databases.

**Fig 2:**
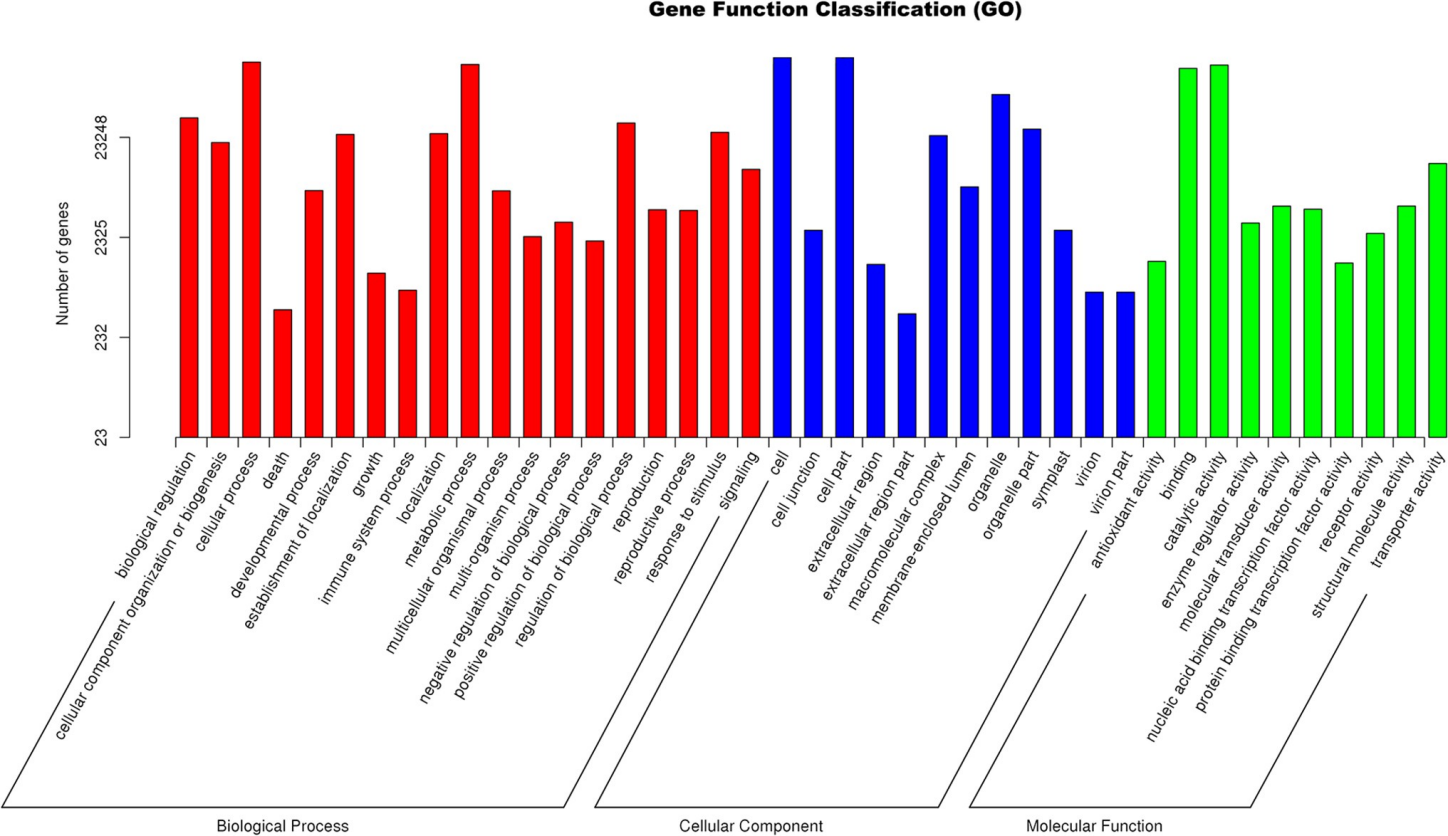
Functional classification of Gene Ontology (GO) for assemble unigenes of *S*. *alopecuroides*. A total of 23,248 unigenes were assigned into 41 functional groups of three categories.

For function classification of the assembled unigenes, 23,248 unigenes were assigned into 41 functional groups of three GO categories which contained the “biological process”, “molecular function”, and “cellular component” (Fig 2). The unigenes classified as being involved in a biological process were shown to have a putative function in the metabolic process (“primary metabolism process”, “response to stimuli”, “biological regulation” and so on). Of the cellular component, “organelle”, “cell”, and “cell part” were the most highly represented terms. Within the molecular function category, “binding”, “catalytic activity”, and “transporter” were highly represented. In addition, the biological pathways and functions of the gene products were analysed according to the KEGG annotations. The mapping of 10,680 (13.63%) unigenes were annotated and classified into 31 biological pathways. It was obvious that three pathways including “translation”, “folding, sorting and degradation”, and “signal transduction” represented the dominant group in *S*. *alopecuroides* (S1 Fig).

### In silico identification of microsatellites based on the transcriptome

In this study, a total of 8,195 putative SSR repeat motifs were identified from 118,197 assembled transcripts, arising from 6.93% of the total number of assembled transcripts. According to the repeat motifs, proper SSR markers primers were designed with strict parameter (S3 Table). Discounting mononucleotide repeats, trinucleotide repeat exhibited the highest frequency of occurrence (49.64%), followed by dinucleotides (46.13%), tetranucleotides (3.71%), pentanucleotides (0.32%) and hexanucleotides (0.21%) (S4 Table). An SSR length of 10 bp was the most abundant, followed by SSR lengths of 15 bp and then of 12 bp (S2 Fig). The most frequent dimer motifs were TC/AG/AT/TA type, followed by CT/GA, whereas for trimeric repeats, GAA/TTC/AGA/TCT/CTT/ AAG were the most abundant (S3 Fig).

### Characterization of polymorphic SSR markers and genetic diversity of *S*. *alopecuroides*

A total of 140 SSR markers were randomly selected and 56 pairs of primers produced good and reproducible banding patterns with expected sizes in the initial screening. Of those successfully amplified primers, only 18 pairs of primers showed higher polymorphism and were used for further genetic diversity and population analysis (Table 1). Except for three SSR loci (SA_SSR7408, SA_SSR7391 and SA_SSR6503), other 15 SSR loci showed HW equilibrium (P > 0.05) (S5 Table). A total of 136 alleles were amplified from the DNA of 260 accessions from different geographic areas. Our results showed that the populations of *S*. *alopecuroides* displayed a relatively high frequency of rare alleles which accounted for 43.38% of total alleles. Interestingly, a considerable number of rare alleles which accounted for 57.23% of total rare allele were detected in five populations (JYN, XY, BC, AWT and LT) consistently located in the southern part of Tianshan Mountains. The number of identified alleles (*N*_a_) ranged from 4 to 8, with an average of 5.91. The number of effective alleles (*N*_e_) varied from 2.51 to 2.97, with an average of 2.81. Across the 18 loci, the observed heterozygosity (*H*_o_) varied from 0.38 to 0.65, with an average of 0.55, whereas the expected heterozygosity (*H*_e_) varied from 0.28 to 0.44, with an average of 0.39. Wright’s fixation index (*F*_is_) varied from—0.65 to—0.23, with an average of—0.38. All statistics of these parameters are summarized in Table 2 and S6 Table.

**Table 1:** Primer sequences, repeat motif, size range of alleles, and annealing temperature (*T*_*m*_) of 18 polymorphic SSR markers developed for *S*. *alopecuroides*.

Primer name	Repeat motif	Primer pair (5’-3’)	Expected size (bp)	Tm (°C)
**SA_SSR7396**	(CTTCA)5	F:TCGACTTGTTTCAATTTCATTCA R:GAGAGTGGCCATCGAAGAAG	270	59
**SA_SSR7403**	(ACAGCA)6	F:GCTCAACAAGCCTTCACCTC R:ACTCATACCCACAGCGGTTC	130	59
**SA_SSR7408**	(TGGCTG)6	F:AAGAAGATGGCGCTGTCACT R: TACATACAGCGGATGGTGGA	209	60
**SA_SSR7405**	(CATCCT)7	F:TCAAAAGTTCCCAGACCAGC R:TGAAGATTTTCACGGTGACG	262	60
**SA_SSR7397**	(GAAAC)5	F:GACCCAGAAGCTCATCAAGC R:CGATTCAAATTCCCACAACC	278	59
**SA_SSR7398**	(GAGAC)5	F:CCACCATTCCATTCACTCCT R:GGAGATGCGGAGAAGTTGAG	151	59
**SA_SSR7391**	(ACGCA)5	F:GACCCCACACATCCCTTCTA R:AATGGGAACACGAAAACGAG	276	59
**SA_SSR7389**	(AAGAT)5	F:TTCAGCTGGTTCCTAGTTCCT R:GCAGCTGCTTTTATTTTTCCC	118	58
**SA_SSR7399**	(GGCGT)5	F:TAATGTTTGGCTGCGAGATG R:CTTTCTTTTTCTTTGCCCCC	166	59
**SA_SSR7400**	(GTGAG)5	F:CACCGTTTGAGTCCATTGTG R:CGAAACCCCTAAAACCCATT	235	60
**SA_SSR7337**	(CTAA)5	F:CATGGATCAAAGGGATGAAGA R:ACGGAAAGCAGTTGGAGAAC	236	59
**SA_SSR7381**	(TTCA)5	F:TAGCAGCCAGTGCTAGTGGA R:AAAGGGAAAGTTGGGAGGAA	116	59
**SA_SSR7376**	(TTAA)5	F:TTGTCAACGCATGACCAAAT R:AGAAGGGTGGAAGCTCACAA	145	59
**SA_SSR6988**	(CTG)8	F:AGTGAATCCAGTGTCTGGGG R:GTGAGTCAAAGAAGGGCTGC	226	60
**SA_SSR7257**	(CTG)7	F:TTCAAACAAGAAGAACCGCC R:GCATGGTTTGCACTCACAAC	227	60
**SA_SSR4793**	(AC)11	F:ACCAATGTTCCCCACCACTA R:AAGGCGGTGAGGTAGCTGTA	275	59
**SA_SSR4947**	(AG)10	F:ACAAGTCTCAACCGTGCCTT R:CTCTTGCTCCTCCGTGAAAC	157	60
**SA_SSR6503**	(CGT)7	F:CCTCGATGACCCAATCTTTG R:AACAAGACGCCGAAGAAGAA	216	60

**Table 2:** Genetic diversity estimates based on 18 SSR loci in 23 populations of *S*. *alopecuroides*.

Populations	*N*_a_	*N*_e_	*I*	*H*_e_	*H*_o_	*F*_is_
**XY**	8	2.51	0.41	0.28	0.38	-0.36
**JYN**	6	2.81	0.57	0.37	0.51	-0.38
**AWT**	7	2.79	0.62	0.39	0.55	-0.41
**LT**	6	2.83	0.63	0.42	0.57	-0.36
**BC**	8	2.88	0.68	0.43	0.56	-0.30
**DQW**	7	2.97	0.72	0.44	0.57	-0.30
**YC**	6	2.93	0.71	0.43	0.57	-0.33
**GMQ**	4	2.78	0.61	0.38	0.54	-0.42
**PL**	6	2.68	0.58	0.34	0.44	-0.29
**ETK**	5	2.97	0.71	0.44	0.65	-0.48
**GJC**	6	2.88	0.67	0.41	0.54	-0.32
**SM**	5	2.92	0.67	0.43	0.59	-0.37
**HSB**	5	2.91	0.67	0.43	0.61	-0.42
**XX**	5	2.81	0.63	0.42	0.63	-0.50
**JY**	6	2.73	0.55	0.37	0.61	-0.65
**DK**	8	2.73	0.56	0.36	0.56	-0.56
**YN**	5	2.86	0.67	0.43	0.57	-0.33
**SPT**	7	2.93	0.68	0.43	0.53	-0.23
**WH**	5	2.82	0.66	0.39	0.56	-0.44
**AZQ**	6	2.77	0.66	0.38	0.47	-0.24
**WY**	4	2.73	0.58	0.36	0.52	-0.44
**BT**	6	2.62	0.49	0.32	0.45	-0.41
**TC**	5	2.77	0.57	0.35	0.45	-0.29
**Mean**	5.91	2.81	0.62	0.39	0.55	-0.38

*N*_a_: Number of Alleles; *N*_e_: No. of Effective Alleles; *I*: Shannon’s Information Index; *H*_o_: Observed heterozygosity; *H*_e_: Expected Heterozygosity; *F*_is_: Fixation Index

### Population differentiation and population structure mirrors geographic structuring pattern of *S*. *alopecuroides*

**Fig 3:**
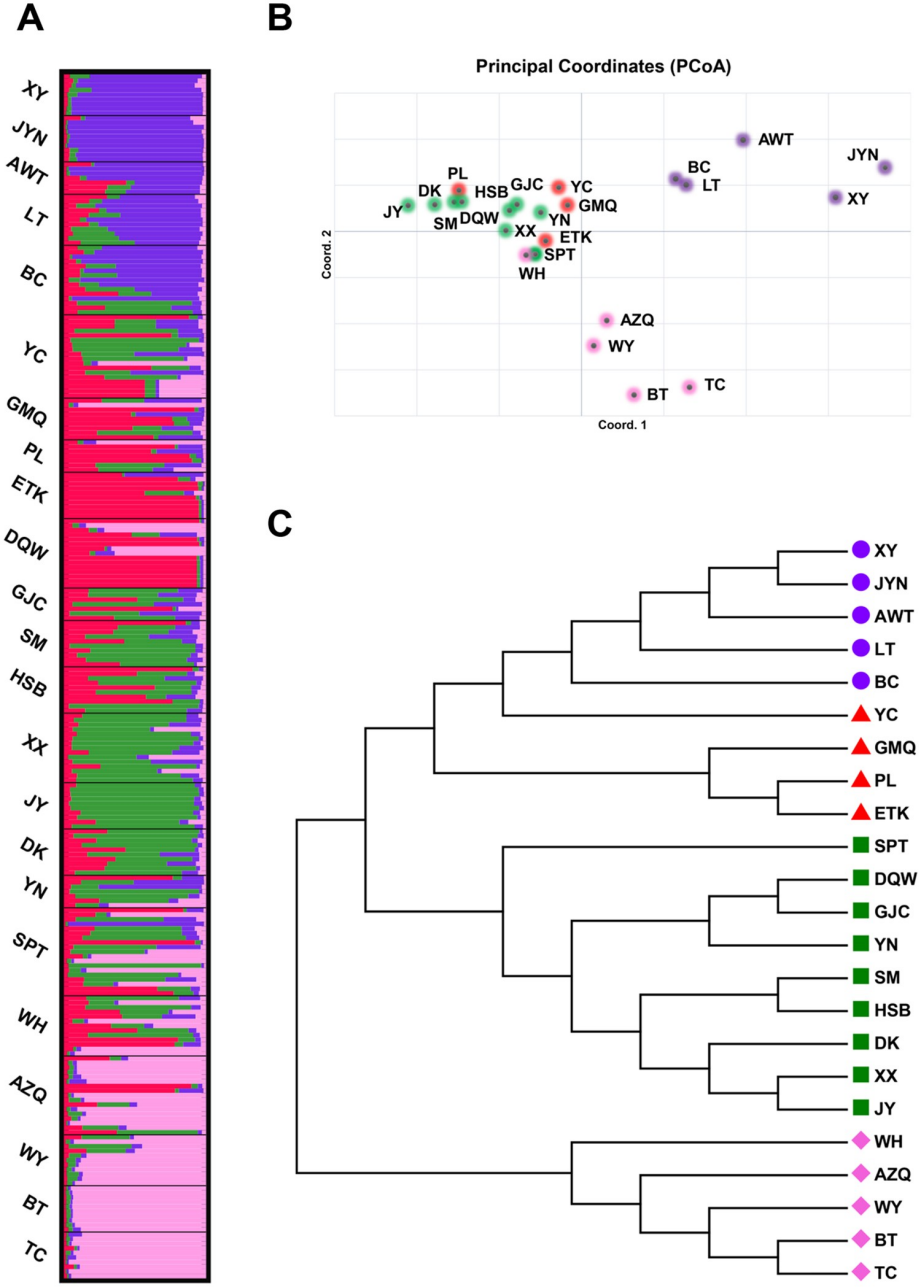
Genetic clustering of all individuals of *S*. *alopecuroides*. (A) Genetic structure results for *K* = 4 using STUCTRUE on a data set of 260 individuals and 18 markers, with replicates averaged by CLUMPP; (B) Principal component analysis (PCoA); (C) Neighbor-joint tree based on the genetic distance. Cluster I, cluster II, cluster III and Cluster IV are presented by blue, pink, red and green color, respectively.

Population structure estimation for the whole panel of accessions was inferred using the Bayesian clustering approach implemented in STRUCTURE version 2.3.4. The most optimal number of clusters was identified using the model value Δ*K* for *K* = 4 (S4 Fig), suggesting that the model with four gene pools captured a major split in the data, clearly showing four geographic clusters (Fig 3A). The results from STRUCTURE produced highly consistent across replicate simulations, which were further confirmed by the among-replicate similarity indice H (a value indicated configuration of individual assignments to each cluster) that was all very close to the maximum of 1 (0.995) according to the calculations by the CLUMPP for the systems with *K* = 4. The first cluster (Cluster I) contained five populations (XY, JYN, AWT, LT and BC) located in the southern part of the Tianshan Mountains. The second cluster (Cluster II) grouped together five populations (TC, BT, WY, WH, and AZQ) collected in the northern part of the Tianshan Mountains. The third cluster (Cluster III) contained four populations (PL, ETK, YC and GMQ) and the fourth cluster (Cluster IV) included nine populations (DQW, GJC, SPT, HSB, XX, YN, DK, JY and SM). Some populations showed mixed distribution between Cluster III and Cluster IV which were located at the Loess Plateau and Inner Mongolia Plateau in the northwest of China. Interestingly, despite the large distance between Cluster II and Cluster III, there were still some populations (AZQ, WH and WY) from the Cluster II mixed with different populations in Cluster III.

PCoA was performed to analyse the genetic relationship and population structure within the 23 natural populations. The first three axes together accounted for 23.17% of the total variation, with 9.17%, 8.90% and 5.10% explained, respectively, by PC axes 1, 2 and 3, respectively. Most populations were divided into four clusters along the circles (Fig 3B) which reveal a high similarity pattern with the population structure mentioned above, although there were some redundant or overlapping accessions. Population structure analysis and PCoA strongly supported the division pattern in *S*. *alopecuroides*, but communicating genetic information also occurred between different clusters. Finally, we found four distinguishable clusters identified according to the unrooted phylogenetic tree constructed using the MEGA programme, consistent with the above-mentioned model-based population structure and PCoA analysis (Fig 3C).

**Fig 4:**
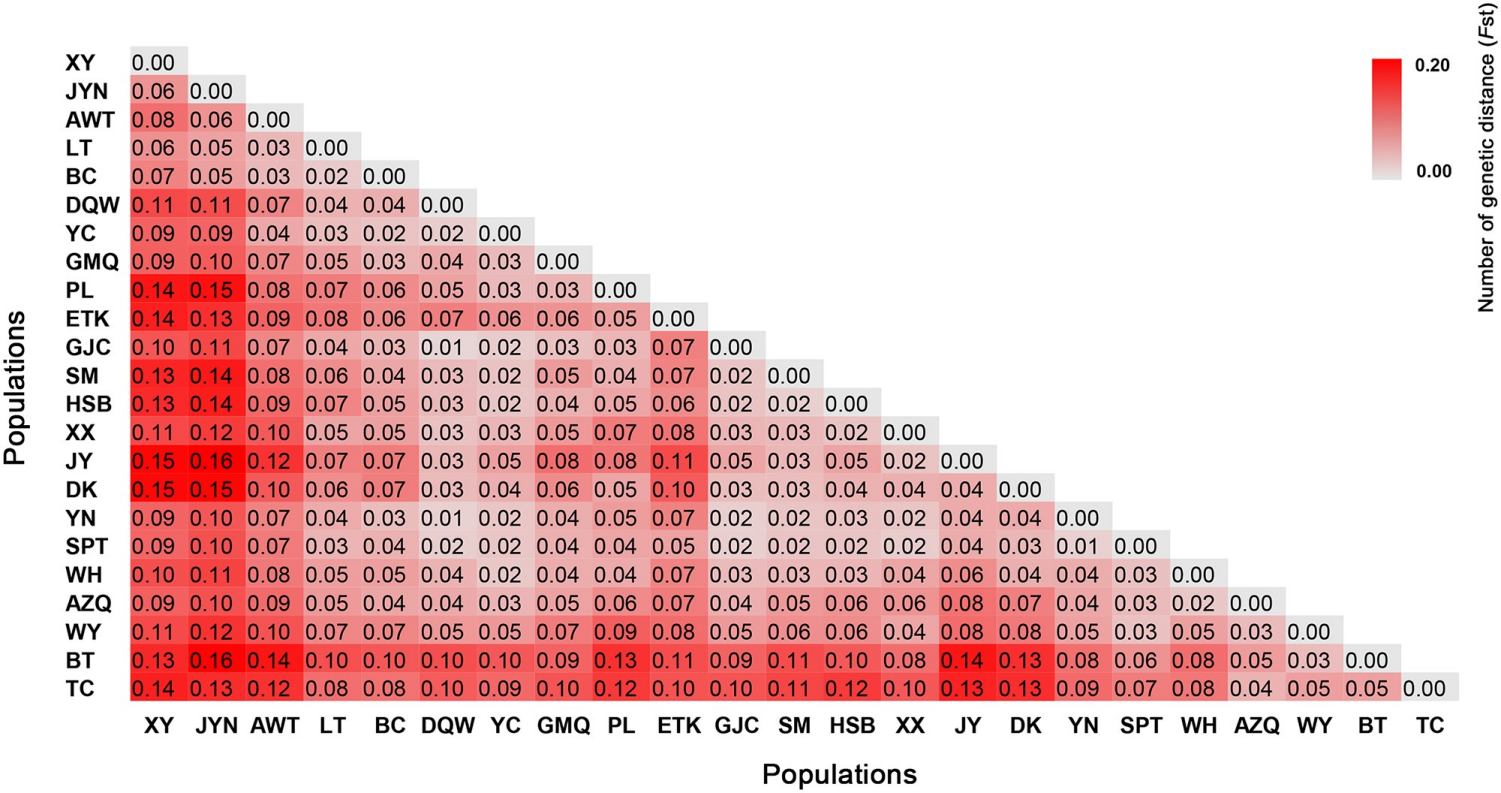
Pairwise population differentiation (*F*_st_) among 23 collection locations of *S*. *alopecuroides* based on the SSR datasets. Cluster I contained five populations (XY, JYN, AWT, LT and BC); Cluster II grouped together five populations (TC, BT, WY, WH, and AZQ); Cluster III contained four populations (PL, ETK, YC and GMQ); Cluster IV included nine populations (DQW, GJC, SPT, HSB, XX, YN, DK, JY and SM).

Additionally, the pairwise genetic differentiation values (*F*_st_) among the 23 populations ranged from 0.01 to 0.16 based on the SSR data (Fig 4). The highest *F*_st_ values were observed in pairwise comparisons between populations from the southern part of Tianshan Mountains (Cluster I, mainly containing population XY, JYN and AWT) and other regions. Beyond that, the AMOVA at the population levels based on sampling locations showed a peculiar pattern. According to the structure analysis, four clusters were considered for AMOVA analysis. It was observed that the genetic diversity due to within-population variation was about 91.98% and the genetic variation among populations accounted for only 8.02% (Table 3).

**Table 3:** Summary analysis of molecular variance.

Source of variation	df	Sum of squares	Variance components	Percentage variation
**Among Pops**	22	182.62	0.23	8.02%
**Within Pops**	497	1357.37	2.72	91.98%
**Total**	519	1539.99	2.95	100%

### Isolation by distance (IBD) and population bottlenecks

The Mantel tests showed a low correlation (r^2^ = 0.2371, p = 0.495), indicating that there was no pattern of isolation by distance among the different populations (S5 Fig). The bottleneck analyses, conducted on the SSR data set, provided support for reduction in genetic variability in *S*. *alopecuroides*. A higher value of *H*_o_ than *H*_e_ observed in 18 markers resulted in negative Wright’s fixation index (*F*_is_) values, indicating a slight excess of heterozygosity in *S*. *alopecuroides*. Excess heterozygosity may be ascribed to a genetic bottleneck. According to the Wilcoxon signed-rank test, all clusters had a significant excess of heterozygosity under the IAM and TPM (*P* < 0.01), whereas a significant bottleneck was only detected in Cluster III and Cluster IV (*P* < 0.01). In addition, mode-shift tests showed that all clusters displayed shifted modes, indicating that *S*. *alopecuroides* experienced a recent bottleneck (Table 4).

**Table 4:** Results of BOTTLENECK tests among populations of *S*. *alopecuroides* based on the Wilcoxon test method and mode-shift test.

Cluster	N	I.A.M	T.P.M	S.M.M	Model
**I**	52	0.00231**	0.00673**	0.06814	Shifted mode
**II**	61	0.00124**	0.00852**	0.10987	Shifted mode
**III**	44	0.00022**	0.00062**	0.00525**	Shifted mode
**IV**	103	0.00012**	0.00036**	0.00306**	Shifted mode

Notes: N = number of individuals; IAM = infinite allele model; TPM = two-phrased model of mutation; SMM = stepwise mutation model;

**, significant (*P* < 0.01)

## Discussion

### Characterization of *S*. *alopecuroides* transcriptome

In recent years, transcriptome sequencing has emerged as an efficient method to generate high-throughput molecular markers output for model or non-model organisms at reasonable cost and speed [37]. To date, however, there is little genetic information on the genome of *S*. *alopecuroides*, which has become an obstacle to deeply understand the molecular mechanism and provide useful strategies to improve its medicinal values, such as alkaloids yield and quality. To our knowledge, we present the first transcriptome assembly described for *S*. *alopecuroides*, which will lay a base for relevant research. According to the annotation results, it was obvious that a large proportion of unigenes showed strong homology to the available sequences in public databases such as Nr, Nt, KOG, KEGG, Swiss-Port and GO. The results obtained here will be better to explore candidate genes or linkage molecular markers for specific agronomic traits, and provide an important genetic resource base for future genetic studies and breeding efforts.

### SSRs as effective markers for assessing the genetic diversity of *S*. *alopecuroides*

Polymorphic microsatellite mining and the genetic variability of SSR markers are now often extensively exploited in non-model plants using a deep transcriptome sequencing approach [16]. However, the relatively limited germplasm collection, no genetic linkage map, lack of appropriate molecular markers of transcriptome or genome sequences have hampered critical research on an assisted breeding programme for *S*. *alopecuroides*, let alone researches into genetic diversity and population structure. We successfully identified a total of 20,324 SSR markers based on transcriptome sequences, and analysis of various motif repeats in *S*. *alopecuroides* revealed that dinucleotides and trinucleotides were the most abundant SSRs (excluding mononucleotide repeats), together accounting for 95.77% of all the SSRs identified.

The characteristics of the SSR markers in *S*. *alopecuroides* were similar to those reported in *S*. *moorcroftiana* [38], another plant in *Sophora*. Moreover, the repeat numbers in the SSR motifs were generally skewed towards fewer repeats with an increase in length of the repeat motif, suggesting that *S*. *alopecuroides* might have undergone a long evolutionary history; Species with long evolutionary histories tend to have an abundance of short motifs [39].

Genetic diversity in a natural population results from the interaction of drift, migration and selection, all of which are necessary for population persistence, adaptation and evolution [40]. It is known that the higher the genetic diversity of a species, the greater its ability to adapt to complicated and changeable environmental conditions over a wide distribution range [41]. *S*. *alopecuroides* has been valued since the earliest times as ecological and economic herbaceous plants, comprehensively utilized for its important role in soil and water conservation across its natural distribution, and unique medicinal properties. Unfortunately, after the field investigation, we found that the limited distribution regions together with the deteriorating ecological environment and human disturbance in the northwest plateau in China, had caused the size of populations and the level of genetic diversity of this species to shrink, which could be further confirmed by the molecular diversity analysis based on the SSR markers (*H*_*o*_ = 0.55, *H*_*e*_ = 0.39) in this study. A total of 18 polymorphic SSR markers were tested and generated alleles for *S*. *alopecuroides*, which provided a relative full assessment of the genetic diversity and population structure of this species for the first time at population level. Compared with other species in the genus *Sophora* [42, 43], such as *S*. *fulvida* (H_*o*_ = 0.44, H_*e*_ = 0.60), *S*. *prostrata* (H_*o*_ = 0.69, H_*e*_ = 0.80), *S*. *molloyi* (H_*o*_ = 0.48, H_*e*_ = 0.72), *S*. *tetraptera* (H_*o*_ = 0.66, H_*e*_ = 0.77), *S*. *microphylla* (H_*o*_ = 0.66, H_*e*_ = 0.79), *S*. *chathamica* (H_*o*_ = 0.54, H_*e*_ = 0.69), *S*. *godleyi* (H_*o*_ = 0.54, H_*e*_ = 0.76), *S*. *longicarinata* (H_*o*_ = 0.58, H_*e*_ = 0.69), *S*. *japonica* (H_*o*_ = 0.74, H_*e*_ = 0.74), it is clear that *S*. *alopecuroides* exhibits relatively low genetic diversity. Besides, most species in *Sophora* are monoecious, insect-pollinated and self-compatible [44]; *S*. *alopecuroides* is no exception, and these factors would lead to its relatively low level of genetic diversity.

### Population structure and genetic differentiation

Combined with the analyses on population structure, PCoA and NJ analyses based on SSR markers, the 23 populations were divided into four distinct genetic clusters significantly related to their geographical origins, further confirmed by the results of hierarchical AMOVA (P < 0.01). From the Mantel test results, we found a low correlation coefficient, r^2^ = 0.2371 (P = 0.495), indicating that geographic distance had very little effect on genetic variance. Although the Mantel test results indicated that the genetic clustering was only very tenuously related to geographical origin of the populations, and only a moderately positive IBD was detected among all *S*. *alopecuroides* samples. Geographic isolation and various climate variation would be major factors that have shaped the current distribution patterns of this species; Many mountains and plateaus resulted in scattered distribution in habitats, which would limit pollen and seed dispersal among populations. Geographic isolation is also expected to have significant effects on the genetic structure of populations, which is a major precursor of allopatric speciation [45, 46]. According to the Bateson-Dobzhansky-Muller model [47], it is expected that in regions where geographic isolation is common in areas where high mountains separate populations occurring in deep valleys, allopatric divergence will be promoted and accelerated due to the formation of locally adapted gene pools. Our findings indicate that the genetic diversity of *S*. *alopecuroides* in the Cluster I was significant differentiated from Cluster II. Numerous mountains and valleys, especially the Tianshan Mountains, constitute geographic barriers between these two groups, thereby leading to the observed high genetic differentiation between them. Coincidentally, an increase number of researches had demonstrated similar phenomenons that many species’ distributions and genetic diversity were deeply influenced by geographical isolation in Northwest China, such as *Gymnocarpos przewalskii* [48], *Hexinia polydichotoma* [49] and *Stuckenia filiformis* [50].

Archaeological evidence and historical records document extensive spread and migration of many plants along the Silk Roads [51], such as apple (*Malus sieversii*), common walnut (*Juglan regia*), peach (*Prunus persica*) and others. In some way, the Silk Road acted as a corridor for species gene flow across topographically diverse landscape and over large distances. Coincidentally, according to our filed investigation, the current distribution areas of *S*. *alopecuroides* are along the famous Silk Road which originated in the historical capital of Shanxi Province (Chang’an), went through Gansu Province via Lanzhou city and Dunhuang city along the Hexi Corridor, and continued westward along the northern foot of the Eastern Tianshan Mountains. Thus, the current spatial of *S*. *alopecuroides* is more likely to be explained by the fact that it was carried as medicine by ancient traders and herders for its anti-inflammatory and analgesic pharmacological effects when they were on a long trip along the Silk Road, and this transport facilitated human-mediated gene flow among autochthonous *S*. *alopecuroides* populations.

### In situ and ex situ conservations implications

Genetic variation plays a crucial role in maintaining a species’ evolutionary potential to cope with continually changing environment. Therefore, maintaining species diversity is one of the main objectives of current species conservation [52, 53]. The present study revealed relatively low genetic diversity levels and high genetic differentiation among populations, which has significant implications for the conservation of endangered species, and for breeding programmes to improve the genetic basis of *S*. *alopecuroides*. Artificial cultivation will be an effective approach to securing supplies for medicinal purposes, generating not only beneficial ecological effects but also considerable economic benefit. Establishing a protection area within the core distribution area where the excavation of *S*. *alopecuroides* was strictly forbidden, which must be implemented immediately for in situ conservation. Several populations from the northern and southern parts of Tianshan Mountains had the higher genetic diversity, which should be prioritized for in situ conservation. A significant number of rare alleles detected in the southern parts of Tianshan Mountains would contribute to *S*. *alopecuroides* breeding, since these rare alleles would be diagnostic for particular genotypes or even have an influence on phenotypic richness. Meanwhile, a germplasm resource nursery or bank through the collection of seeds from different geographic locations should be established urgently. Furthermore, improving the genetic diversity within populations requires the monitoring of crossing among populations. When ex situ conservation is implemented, sample collection should cover as many populations as possible from the entire natural range to capture the full extent of genetic variability. In conclusion, combining in situ and ex situ conservation approaches would be the best strategy to preserve the valuable genetic resources of *S*. *alopecuroides*.

## Conclusion

A large-scale transcriptome dataset from *S*. *alopecuroides* with 118,197 assembled transcripts of mean length 735 bp was generated and a total of 18 markers were validated in 260 accessions from 23 populations. As a result, genetic diversity was discovered to be relatively low, mainly due to the deteriorating ecological environment and declining population numbers of *S*. *alopecuroides*. The complex geographic distribution divided *S*. *alopecuroides* into four distinctive genetic clusters. Geographical isolation together with human migration and trade along the Silk Road would influence the distribution of *S*. *alopecuroides* and promote the spread of *S*. *alopecuroides* from the western to eastern part of the northwest plateau in China. To the best of our knowledge, this is the first attempt to exploit a transcriptome database and develop a set of SSR markers in *S*. *alopecuroides*, a resource that will facilitate in-depth understanding of genetic diversity and population genetic structure and also promote researches on evolution ecology, conservation genetics and genetic breeding of *S*. *alopecuroides* in the future.

## Supporting information

S1 Fig Function classification of unigenes of *S*. *alopecuroides* in KEGG category. Total of 10,680 unigenes were assigned into 31 functional groups of five cluster.
(TIF)

S2 Fig The frequency of the length of repeat motifs in *S*. *alopecuroides*. (TIF)

S3 Fig Types and frequency of most abundant repeat motifs in *S*. *alopecuroides* (low abundant motif not displayed). (TIFF)

S4 Fig Determination of the optimal number (*K*) of populations for *S*. *alopecuroides* according to the Evanno’s admixture analysis. (TIF)

S5 Fig Relationships between genetic distances (pairwise *F*_st_) and geographic distance for a 23 sampled populations of *S*. *alopecuroides*. Genetic and geographical distances showed low correlation.
(TIF)

S1 Table Geographic locations and sample sizes of *S*. *alopecuroides* in China. (DOCX)

S2 Table Summary statistics for the de novo assembled transcriptome of *S*. *alopecuroides*. (DOCX)

S3 Table Distribution to different repeat classes of SSR motifs in *S*. *alopecuroides*. (XLS)

S4 Table All SSR primer pairs of *S*. *alopecuroides* based on the transcriptome. (DOCX)

S5 Table Tests for Hardy-Weinberg equilibrium of 18 SSR loci among 260 *S*. *alopecuroides* accessions. (DOCX)

S6 Table Characteristics of 18 SSR loci in a collection of 260 *S*. *alopecuroides* accessions. (DOCX)
