# Supplementary figures and images for: The genetic diversity and population structure of Sophora alopecuroides (Faboideae) as determined by microsatellite markers developed from transcriptome
Source: PLoS One. 2019 Dec 5;14(12):e0226100. doi: 10.1371/journal.pone.0226100 (PMC6894834; doi:10.1371/journal.pone.0226100)

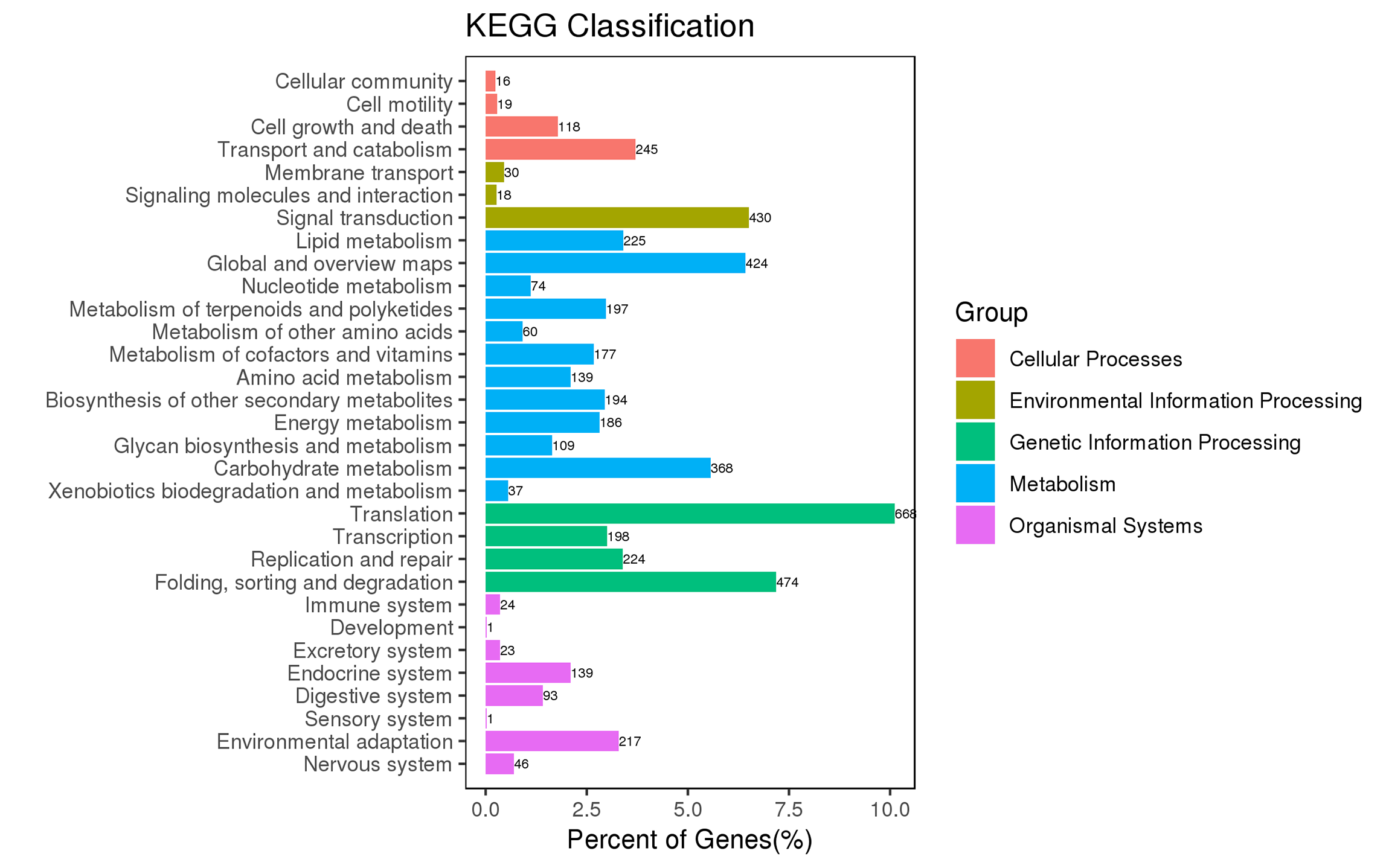

Supplement: S1 Fig — Total of 10,680 unigenes were assigned into 31 functional groups of five cluster. (TIF) [file pone.0226100.s001.tif]

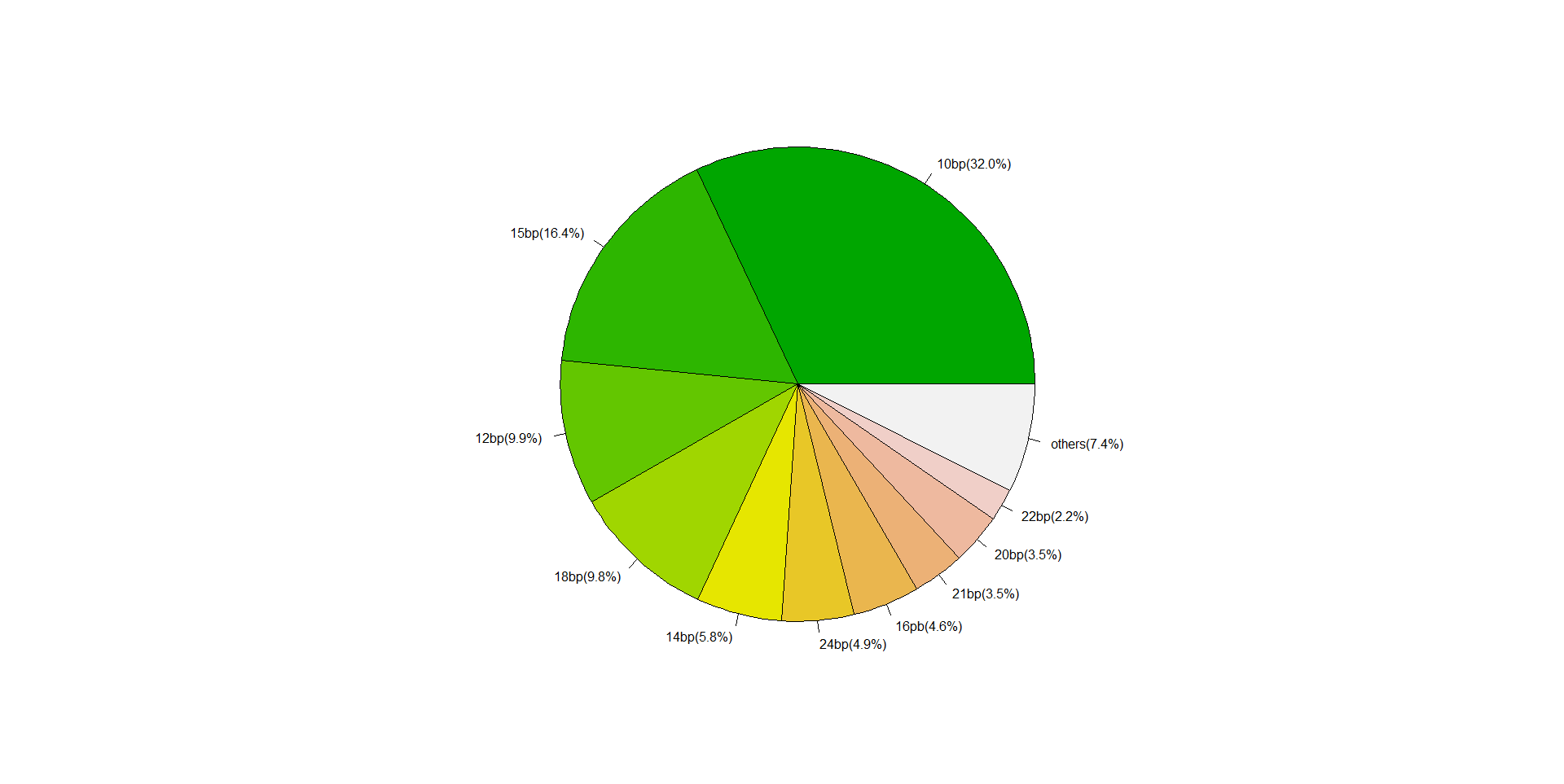

Supplement: S2 Fig — (TIF) [file pone.0226100.s002.tif]

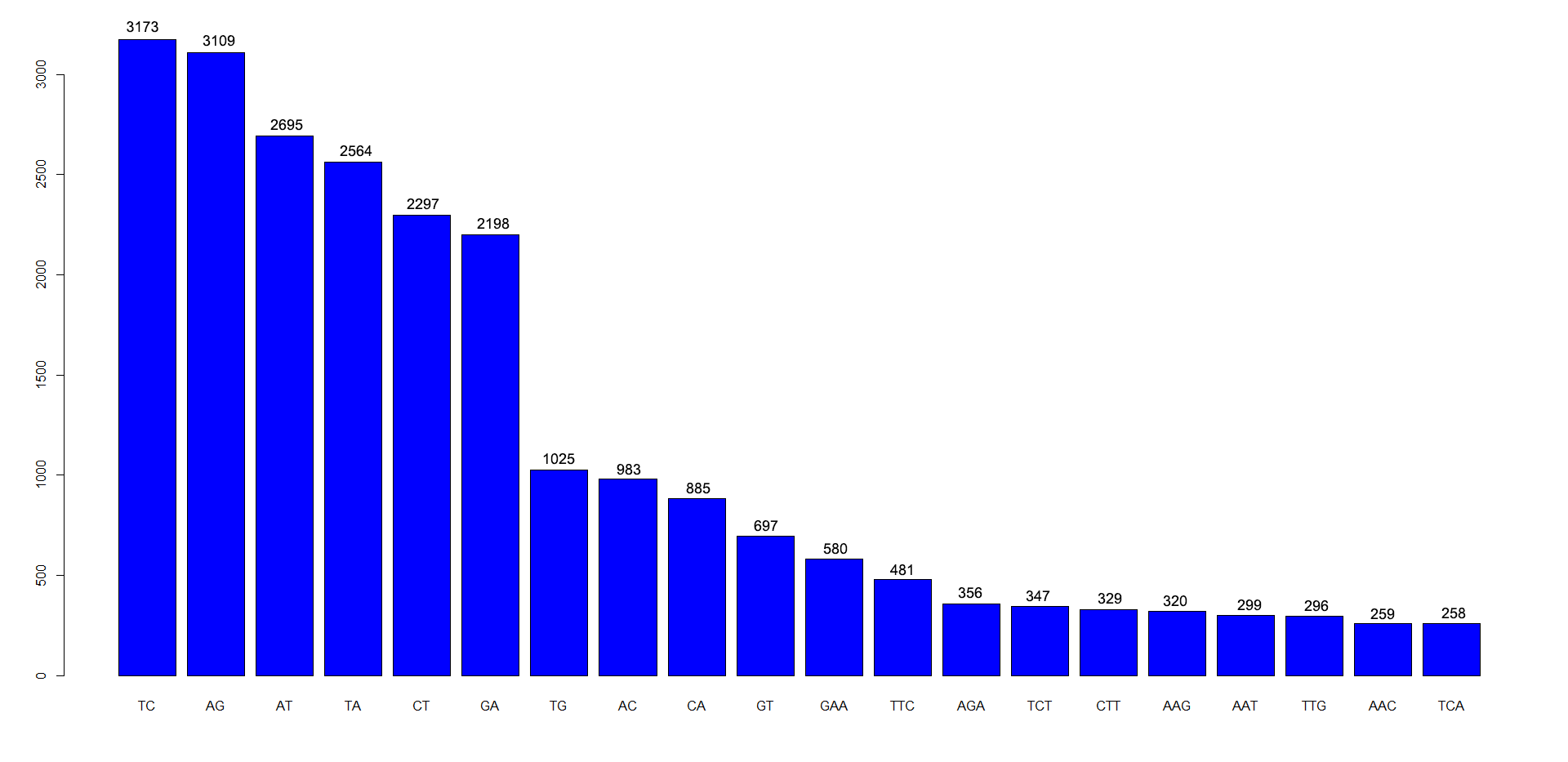

Supplement: S3 Fig — (TIFF) [file pone.0226100.s003.tiff]

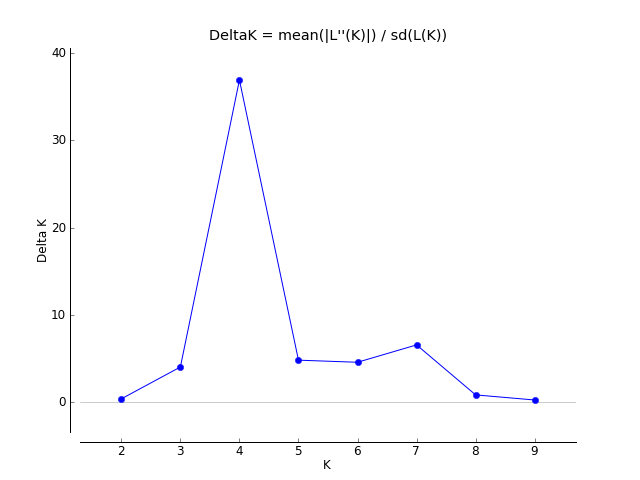

Supplement: S4 Fig — (TIF) [file pone.0226100.s004.tif]

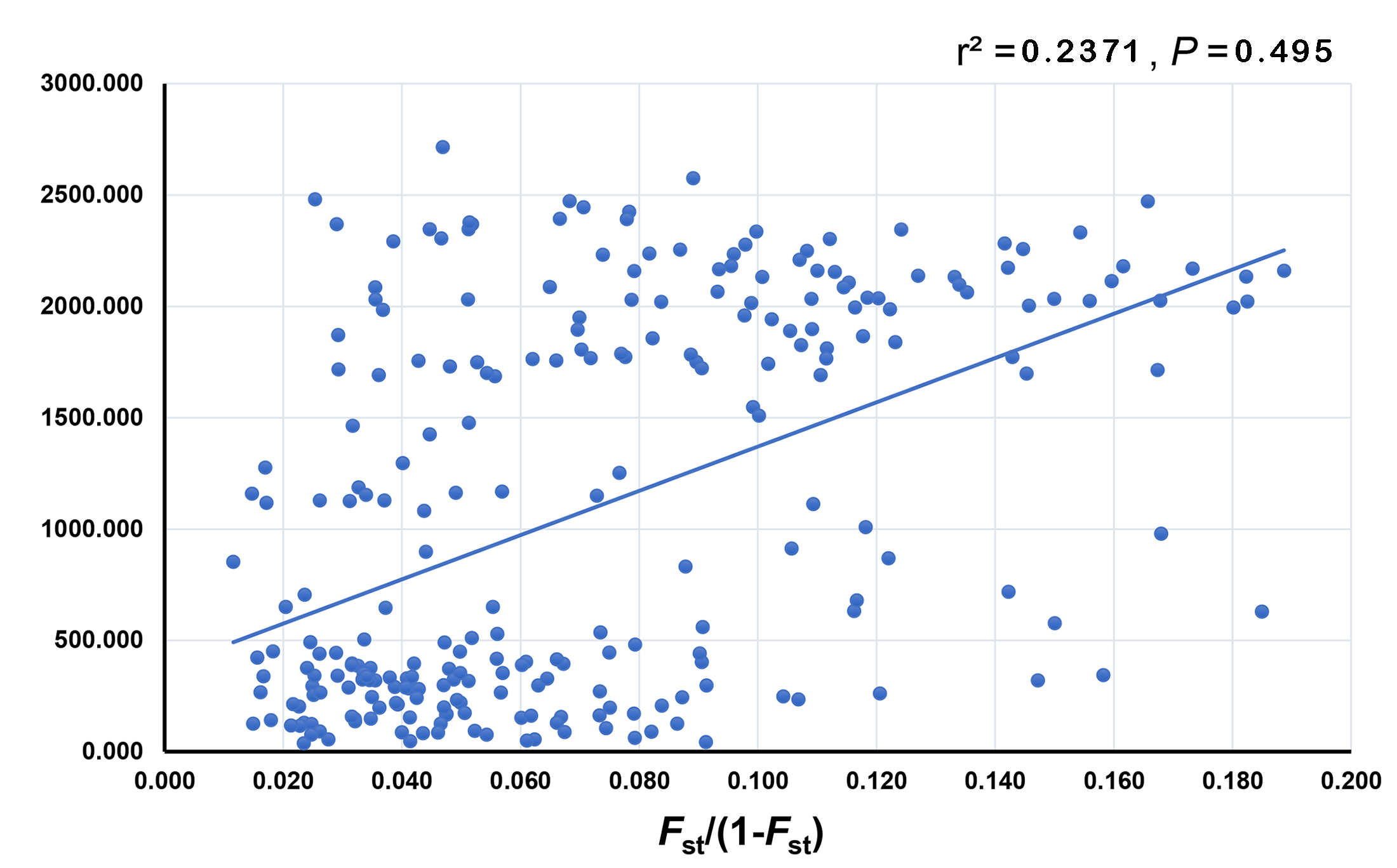

Supplement: S5 Fig — Genetic and geographical distances showed low correlation. (TIF) [file pone.0226100.s005.tif]
