# Supplementary material for: The genetic diversity and population structure of Sophora alopecuroides (Faboideae) as determined by microsatellite markers developed from transcriptome
Source: PLoS One. 2019 Dec 5;14(12):e0226100. doi: 10.1371/journal.pone.0226100 (PMC6894834; doi:10.1371/journal.pone.0226100)
Supplement: S1 Table — (DOCX) [file pone.0226100.s006.docx]

| **S1 Table. Geographic locations and sample sizes of *S. alopecuroides* in China** | | | | | |
| --- | --- | --- | --- | --- | --- |
| **Code Name** | **Region of origin** | **Longitude(E)** | **Latitude(N)** | **Altitude(m)** | **Sample size** |
| **XY** | Xinyuan, Xinjiang Uyghur Autonomous Region | 83°14' | 43°24' | 985 | 9 |
| **JYN** | Yining, Xinjiang Uyghur Autonomous Region | 81°32' | 44°00' | 887 | 10 |
| **AWT** | Awati, Xinjiang Uyghur Autonomous Region | 80°23' | 40°28' | 1036 | 7 |
| **LT** | Luntai, Xinjiang Uyghur Autonomous Region | 86°08' | 41°47' | 1012 | 11 |
| **BC** | Bachu, Xinjiang Uyghur Autonomous Region | 78°49' | 39°53' | 1105 | 15 |
| **DQW** | Daquanwan, Xinjiang Uyghur Autonomous Region | 93°53' | 42°43' | 815 | 15 |
| **YC** | Yanchi, Ningxia Hui Autonomous Region | 107°25' | 37°44' | 1344 | 18 |
| **GMQ** | Minqin, Gansu Province | 103°01' | 38°35' | 1382 | 9 |
| **PL** | Pingluo, Ningxia Hui Autonomous Region | 106°42' | 38°49' | 1102 | 7 |
| **ETK** | Etuokeqianqi, Inner Mongolia Autonomous Region | 107°28' | 38°14' | 1321 | 10 |
| **GJC** | Jinchang, Gansu Province | 102°23' | 38°34' | 1414 | 7 |
| **SM** | Shenmu, Shanxi Province | 110°30' | 38°49' | 1038 | 10 |
| **HSB** | Hongsibao, Ningxia Hui Autonomous Region | 106°00' | 37°26' | 1325 | 10 |
| **XX** | Xixia, Ningxia Hui Autonomous Region | 106°05' | 38°33' | 1126 | 15 |
| **JY** | Jiuyuan, Inner Mongolia Autonomous Region | 107°28' | 40°35' | 1018 | 10 |
| **DK** | Dengkou,Inner Mongolia Autonomous Region | 107°01' | 40°19' | 1053 | 10 |
| **YN** | Yongning, Ningxia Autonomous Region | 106°15' | 38°14' | 1118 | 7 |
| **SPT** | Shapotou, Ningxia Autonomous Region | 105°08' | 37°31' | 1230 | 19 |
| **WH** | Haibowan, Inner Mongolia Autonomous Region | 106°42' | 39°34' | 1079 | 13 |
| **AZQ** | Azuoqi, Inner Mongolia Autonomous Region | 105°41' | 38°52' | 1561 | 17 |
| **WY** | Wuyuan, Inner Mongolia Autonomous Region | 107°46' | 40°54' | 1035 | 11 |
| **BT** | Beitun, Xinjiang Autonomous Region | 88°08' | 47°17' | 548 | 10 |
| **TC** | Emin, Xinjiang Autonomous Region | 83°37' | 46°29' | 509 | 10 |
