# Supplementary material for: The genetic diversity and population structure of Sophora alopecuroides (Faboideae) as determined by microsatellite markers developed from transcriptome
Source: PLoS One. 2019 Dec 5;14(12):e0226100. doi: 10.1371/journal.pone.0226100 (PMC6894834; doi:10.1371/journal.pone.0226100)
Supplement: S2 Table — (DOCX) [file pone.0226100.s007.docx]

| **S2 Table. Summary statistics for the de novo assembled transcriptome of S. alopecuroides** | |
| --- | --- |
| **Category** | **Numbers** |
| **Total Clean Reads** | 390,247,286 |
| **Maximum sequence length (bp)** | 16,303 |
| **Minimum sequence length (bp)** | 200 |
| **Average sequence length (bp)** | 735 |
| **GC content (%)** | 47.96 |
| **N50 length** | 1,213 |
| **Total number of sequences examined** | 118,197 |
| **Total size of examined sequences (bp)** | 87,686,071 |
| **Total number of identified SSRs** | 20,324 |
| **Number of SSR containing sequences** | 17,350 |
| **Number of sequences containing more than one SSR** | 2,497 |
