# Supplementary material for: The genetic diversity and population structure of Sophora alopecuroides (Faboideae) as determined by microsatellite markers developed from transcriptome
Source: PLoS One. 2019 Dec 5;14(12):e0226100. doi: 10.1371/journal.pone.0226100 (PMC6894834; doi:10.1371/journal.pone.0226100)
Supplement: S4 Table — (DOCX) [file pone.0226100.s009.docx]

**S4 Table. Distribution to different repeat classes of SSRs motifs.**

| **Repeats** | **5** | **6** | **7** | **8** | **9** | **>=10** | **Total** | **%** |
| --- | --- | --- | --- | --- | --- | --- | --- | --- |
| **Dinucleotide** | 0 | 1280 | 774 | 612 | 438 | 676 | 3780 | 46.13 |
| **Trinucleotide** | 2363 | 1124 | 550 | 23 | 0 | 8 | 4068 | 49.64 |
| **Tetranucleotide** | 245 | 49 | 4 | 2 | 2 | 2 | 304 | 3.71 |
| **Pentanucleotide** | 23 | 0 | 2 | 0 | 1 | 0 | 26 | 0.32 |
| **Hexanucleotide** | 7 | 8 | 2 | 0 | 0 | 0 | 17 | 0.21 |
| **Total** | 2638 | 2461 | 1332 | 637 | 441 | 686 | 8195 | 100 |
