# Supplementary material for: The genetic diversity and population structure of Sophora alopecuroides (Faboideae) as determined by microsatellite markers developed from transcriptome
Source: PLoS One. 2019 Dec 5;14(12):e0226100. doi: 10.1371/journal.pone.0226100 (PMC6894834; doi:10.1371/journal.pone.0226100)
Supplement: S5 Table — (DOCX) [file pone.0226100.s010.docx]

**S5 Table. Tests for Hardy-Weinberg Equilibrium of 18 SSR loci among 260 *S. alopecuroides* accessions**

| **SSR Locus** | **ChiSquare** | **DF** | ***P* value** |
| --- | --- | --- | --- |
| SA_SSR7396 | 0.037 | 2 | 0.321 |
| SA_SSR7403 | 0.083 | 3 | 0.433 |
| SA_SSR7408* | 0.021 | 1 | 0.023 |
| SA_SSR7405 | 0.365 | 3 | 0.292 |
| SA_SSR7397 | 0.159 | 3 | 0.079 |
| SA_SSR7398 | 0.582 | 3 | 0.407 |
| SA_SSR7391* | 0.042 | 1 | 0.002 |
| SA_SSR7389 | 0.511 | 4 | 0.577 |
| SA_SSR7399 | 0.421 | 1 | 0.292 |
| SA_SSR7400 | 0.025 | 6 | 0.067 |
| SA_SSR7337 | 0.079 | 2 | 0.468 |
| SA_SSR7381 | 0.027 | 1 | 0.071 |
| SA_SSR7376 | 0.311 | 3 | 0.577 |
| SA_SSR6988 | 0.052 | 2 | 0.063 |
| SA_SSR7257 | 0.089 | 1 | 0.095 |
| SA_SSR4793 | 0.075 | 3 | 0.088 |
| SA_SSR4947 | 0.045 | 1 | 0.078 |
| SA_SSR6503* | 0.036 | 2 | 0.031 |

Note: The *P* value greater than 0.05 indicated that the locus showed HW equilibrium.
