# Supplementary material for: The genetic diversity and population structure of Sophora alopecuroides (Faboideae) as determined by microsatellite markers developed from transcriptome
Source: PLoS One. 2019 Dec 5;14(12):e0226100. doi: 10.1371/journal.pone.0226100 (PMC6894834; doi:10.1371/journal.pone.0226100)
Supplement: S6 Table — (DOCX) [file pone.0226100.s011.docx]

**S6 Table. Characteristics of 18 SSR loci in a collection of 260 *S. alopecuroides* accessions**

| **Locus** | ***N*_a_** | ***N*_e_** | ***N*_r_** | ***N*_u_** | ***H*_o_** | ***H*_e_** | **PIC** |
| --- | --- | --- | --- | --- | --- | --- | --- |
| **SA_SSR7396** | 8 | 2.6 | 4 | 3 | 0.19 | 0.42 | 0.52 |
| **SA_SSR7403** | 7 | 3.7 | 2 | 1 | 0.37 | 0.51 | 0.41 |
| **SA_SSR7408** | 9 | 2.1 | 5 | 0 | 0.33 | 0.57 | 0.62 |
| **SA_SSR7405** | 8 | 3.4 | 3 | 1 | 0.42 | 0.56 | 0.54 |
| **SA_SSR7397** | 6 | 2.3 | 3 | 0 | 0.31 | 0.55 | 0.43 |
| **SA_SSR7398** | 8 | 3.7 | 3 | 1 | 0.35 | 0.58 | 0.51 |
| **SA_SSR7391** | 6 | 1.5 | 2 | 1 | 0.21 | 0.38 | 0.39 |
| **SA_SSR7389** | 8 | 2.2 | 3 | 1 | 0.43 | 0.57 | 0.53 |
| **SA_SSR7399** | 9 | 3.6 | 4 | 1 | 0.35 | 0.61 | 0.59 |
| **SA_SSR7400** | 7 | 1.3 | 4 | 1 | 0.29 | 0.42 | 0.50 |
| **SA_SSR7337** | 6 | 2.5 | 2 | 0 | 0.23 | 0.49 | 0.41 |
| **SA_SSR7381** | 8 | 2.7 | 3 | 0 | 0.26 | 0.53 | 0.58 |
| **SA_SSR7376** | 8 | 2.2 | 4 | 0 | 0.36 | 0.55 | 0.57 |
| **SA_SSR6988** | 7 | 3.7 | 2 | 1 | 0.39 | 0.61 | 0.62 |
| **SA_SSR7257** | 9 | 1.4 | 5 | 2 | 0.38 | 0.59 | 0.51 |
| **SA_SSR4793** | 8 | 4.3 | 4 | 1 | 0.45 | 0.75 | 0.61 |
| **SA_SSR4947** | 9 | 2.6 | 4 | 3 | 0.35 | 0.72 | 0.58 |
| **SA_SSR6503** | 5 | 2.2 | 2 | 0 | 0.25 | 0.47 | 0.40 |

*N*_a_: number of alleles; *N*_e_: effective alleles; *N*_r_: rare alleles; *N*_u_: unique alleles; *H*_o_: observed heterozygosity; *H*_e_: expected heterozygosity; PIC: polymorphic information content
